# Immune Checkpoint Inhibitor-Related Pneumonitis in Renal Cell Carcinoma: Clinical Features, Mechanisms, and Lessons from Lung Cancer

**DOI:** 10.3390/biom16081117

**Published:** 2026-07-30

**Authors:** Kristian Shtembari, Martina Catalano, Ismaela Anna Vascotto, Chiara Calandrelli, Silvia Mancini, Luca Pratesi, Martina Izzi, Marinella Micol Mela, Virginia Rossi, Serena Pillozzi, Alejo Rodriguez-Vida, Mohamed Aseafan, Maria Tereza Nieto-Coronel, Matteo Santoni, Lorenzo Antonuzzo, Giandomenico Roviello

**Affiliations:** 1 Department of Experimental and Clinical Medicine, University of Florence, 50134 Florence, Italyluca.pratesi@unifi.it (L.P.);; 2 Department of Health Sciences, Section of Clinical Pharmacology and Oncology, University of Florence, Viale Pieraccini, 6, 50139 Florence, Italy; martina.catalano@unifi.it; 3 Clinical Oncology, Careggi University Hospital, 50134 Florence, Italy; 4 Hospital del Mar, 08003 Barcelona, Spain; 5 Section of Medical Oncology, Department of Internal Medicine, Security Forces Hospital, Riyadh 11481, Saudi Arabia; 6 College of Medicine, Alfaisal University, Riyadh 11533, Saudi Arabia; 7 Oncopalia, MyA Center, Medical Oncology Departament, La Paz City P.O. Box 8635, Bolivia; 8 Oncology Unit, Macerata Hospital, 62100 Macerata, Italy

**Keywords:** renal cell carcinoma, immune checkpoint inhibitors, immune-related adverse events, pneumonitis, checkpoint inhibitor-related pneumonitis, tyrosine kinase inhibitors, macrophage pyroptosis, GSDME, steroid-refractory pneumonitis, immunotherapy toxicity

## Abstract

Immune checkpoint inhibitor-related pneumonitis (CIP) is an uncommon but clinically relevant toxicity in renal cell carcinoma (RCC), where immune checkpoint inhibitors are frequently used either as dual immunotherapy or in combination with VEGF-targeted tyrosine kinase inhibitors. In RCC, CIP poses specific diagnostic challenges because respiratory symptoms and computed tomography findings may overlap with pulmonary metastases, infections, thromboembolic events, heart failure, and TKI-related lung toxicity. This review summarizes the incidence of CIP across pivotal RCC trials and real-world cohorts, compares its epidemiology with non-small cell lung cancer, and discusses clinical presentation, radiological patterns, differential diagnosis, and current management strategies. Particular attention is given to RCC-specific mechanisms, including immune-mediated alveolar injury, T cell activation, cytokine dysregulation, macrophage activation, GSDME-mediated pyroptosis, and the potential contribution of VEGF pathway inhibition to pulmonary inflammation. We also review risk factors, steroid-refractory disease, second-line immunosuppression, and the unresolved issue of ICI rechallenge after pneumonitis. A better understanding of these mechanisms and clinical features may improve early recognition, guide multidisciplinary management, and support safer use of immunotherapy-based combinations in patients with RCC.

## 1. Introduction

Over the past decade, immune checkpoint inhibitors (ICIs) have profoundly reshaped the therapeutic landscape of advanced renal cell carcinoma (RCC). By targeting inhibitory immune pathways such as PD-1, PD-L1 and CTLA-4, ICIs have expanded treatment strategies beyond conventional targeted therapies and enabled durable responses in a subset of patients [1,2,3]. Initial successes with monotherapy have led to more intensive approaches, including dual ICI regimens and, more recently, combinations pairing anti-PD-(L)1 agents with VEGF-targeted tyrosine kinase inhibitors (TKIs) [4,5,6,7]. These regimens exploit complementary mechanisms, namely immune activation together with angiogenesis modulation, and they now represent key first-line treatment options. As a result, a growing proportion of patients with metastatic RCC are exposed to ICIs early in their treatment course and often for prolonged durations.

With the expanding use of immunotherapy, increasing attention has focused on immune-related adverse events (irAEs), a heterogeneous group of toxicities arising from off-target immune activation. irAEs may involve nearly any organ system, and their incidence and severity vary according to drug class, treatment combination and individual susceptibility [8,9]. They have become critical determinants of treatment tolerance, quality of life and, in some cases, clinical outcomes. In RCC, where long-term disease control is possible for selected patients, timely recognition and management of irAEs are essential to maintain therapeutic benefit while minimizing morbidity. Among these toxicities, immune checkpoint inhibitor-related pneumonitis (CIP) represents one of the most challenging.

CIP is an uncommon but potentially life-threatening toxicity characterized by immune-mediated inflammation of the lung parenchyma. Although it occurs less frequently than many other irAEs, it carries a relatively disproportionately high risk of severe complications, including respiratory failure and death. Early diagnosis is often difficult because symptoms such as cough, dyspnea and fever are nonspecific, while radiologic findings may overlap with a wide range of pulmonary conditions. These challenges are particularly relevant in RCC, where pulmonary metastases, lung toxicity related to TKIs, infectious complications and pre-existing lung disease may mimic or mask CIP. As ICI and TKI combinations become increasingly common, the interaction between immune-mediated and drug-induced pulmonary effects adds further complexity to clinical evaluation.

Most of the current knowledge on CIP comes from studies in non-small cell lung cancer (NSCLC) [10,11,12]. In this setting, higher baseline pulmonary vulnerability caused by tumor location, smoking-related comorbidities and prior thoracic treatments contributes to an increased incidence. NSCLC studies have provided detailed descriptions of clinical and radiologic features as well as management outcomes, and these data inform current guidelines [13,14]. However, the direct applicability of these findings to RCC remains uncertain. Because RCC-specific data remain relatively limited, several concepts discussed in this review are necessarily informed by evidence derived from NSCLC and mixed solid-tumor populations. Whenever possible, RCC-specific findings are presented separately from extrapolated evidence to highlight both established knowledge and current gaps in the literature. Differences in tumor biology, metastatic behavior and therapeutic strategies suggest that CIP in RCC may present with distinct risk factors, frequencies and prognostic implications. The limited availability of RCC-specific data, especially in the context of ICI and TKI combinations now widely used, highlights a relevant unmet clinical need.

Given the expanding integration of ICIs into RCC therapy and the growing complexity of treatment regimens, a more refined understanding of CIP in this disease context is urgently required. This review synthesizes available evidence on the incidence, presentation, mechanisms and management of CIP in RCC, drawing comparisons with NSCLC where appropriate. By identifying current knowledge gaps and proposing areas for future research, we aim to support earlier recognition, more accurate diagnosis and safer therapeutic strategies for patients at risk of this underappreciated yet clinically significant toxicity.

Literature for this narrative review was identified through searches of PubMed/MEDLINE and Google Scholar using combinations of the following keywords: “renal cell carcinoma”, “immune checkpoint inhibitors”, “pneumonitis”, “immune-related adverse events”, “PD-1”, “PD-L1”, “CTLA-4”, and “tyrosine kinase inhibitors”. Priority was given to pivotal clinical trials, meta-analyses, consensus guidelines, and relevant translational studies. Additional references were identified through manual review of the bibliographies of selected articles. Given the narrative nature of the review, no formal systematic inclusion or exclusion criteria were applied.

## 2. Epidemiology and Incidence

The epidemiology of immune checkpoint inhibitor-related pneumonitis (CIP) in renal cell carcinoma (RCC) remains incompletely characterized, owing to restrictive eligibility criteria in pivotal clinical trials, heterogeneous toxicity reporting and its low incidence [15]. Nevertheless, phase III trials establishing immune checkpoint inhibitors (ICIs) as standard therapy in advanced RCC—such as CheckMate 214 (nivolumab + ipilimumab), KEYNOTE-426 (pembrolizumab + axitinib), and CLEAR (lenvatinib + pembrolizumab)—offer key insights into CIP frequency and severity across monotherapy, dual-ICI, and ICI–tyrosine kinase inhibitor (TKI) combinations. All-grade CIP rates typically range from 3% to 5% in these trials, though real-world evidence reveals higher incidences (up to 8–10%) and greater clinical heterogeneity [16,17]. Comparisons with non-small cell lung cancer (NSCLC), where CIP is more common and more extensively studied, further contextualize the epidemiologic landscape and highlight the need for RCC-specific surveillance strategies.

### 2.1. CIP Incidence in Pivotal Clinical Trials of RCC

The incidence of CIP in pivotal RCC trials varies by regimen, with dual immune checkpoint inhibitor (ICI) combinations showing higher rates than ICI–tyrosine kinase inhibitor (TKI) therapies. Table 1 summarizes key data. Importantly, incidence estimates reported in pivotal clinical trials should be interpreted cautiously. Trial populations are highly selected and frequently exclude patients with significant pulmonary comorbidities, while pneumonitis reporting often relies on investigator assessment without centralized radiologic adjudication. Consequently, real-world studies generally report higher incidence rates and greater heterogeneity than registration trials.

Dual-ICI regimens like nivolumab + ipilimumab carry the highest CIP risk due to intensified immune activation, consistent across tumor types [20,21]. In contrast, ICI–TKI combinations exhibit lower rates but unique challenges, including diagnostic overlap with TKI-related pulmonary toxicity or infection.

A key limitation across trials is investigator-reported outcomes, which often underestimate true incidence through misattribution to infection, progression, or TKI effects—particularly without standardized radiology or protocols [15,22,23]. Trial exclusions of patients with pre-existing lung disease may further artificially increase the apparent safety profile.

### 2.2. Differences Across Monotherapy, Dual-ICI and ICI–TKI Regimens

The risk of CIP is influenced by both the intensity and type of immune activation-induced by ICIs. Monotherapy with PD-1 or PD-L1 inhibitors, such as nivolumab or pembrolizumab, generally carries the lowest risk, with pneumonitis typically occurring in fewer than 3 percent of patients (2.7%, with ranges from 1.3% to 3.6% depending on PD-1 vs. PD-L1 agents) [18,24,25,26,27]. These findings are consistent across tumor types and likely reflect the more selective mechanism of PD-1/PD-L1 blockade, which primarily modulates exhausted T cell function without broadly amplifying T cell priming [12,20].

Dual-ICI therapy, particularly the combination of nivolumab and ipilimumab, is associated with a higher incidence of CIP (10% incidence vs. 3% for monotherapy (*p* < 0.001). CTLA-4 inhibition increases early T cell priming and broadens the immune repertoire, which may potentiate immune-mediated inflammation in the lung parenchyma. As such, CIP rates in dual-ICI regimens often exceed those of monotherapy by two- to threefold (RR 3.47 (95% CI 1.76–6.83) for all-grade pneumonitis with nivolumab plus ipilimumab vs. monotherapy), and severe events requiring hospitalization or high-dose corticosteroids are more frequent. The pattern observed in RCC mirrors that seen in melanoma and NSCLC, supporting a mechanistic basis for higher pneumonitis risk with combined checkpoint blockade [20,26].

ICI–TKI combinations occupy an “intermediate position” [28,29]. Although the immunologic stimulation from PD-1/PD-L1 inhibition remains significant, the addition of VEGF-targeted TKIs may alter pulmonary vascular permeability and immune cell trafficking in ways that modulate, but do not necessarily exacerbate, the risk of CIP. However, the coexistence of TKI-related pulmonary effects can complicate clinical interpretation. For example, axitinib, lenvatinib and cabozantinib have each been associated with rare pulmonary adverse events, including interstitial lung disease, pleural effusions or dyspnea [30,31]. Distinguishing these toxicities from true CIP is challenging and often necessitates careful clinical correlation, high-resolution computed tomography (HRCT), and, in selected cases, bronchoscopy with bronchoalveolar lavage.

### 2.3. Real-World Incidence: Higher Variability and Diagnostic Uncertainty

Real-world studies provide a more comprehensive view on the epidemiology of CIP in RCC. Across observational cohorts, the reported incidence of pneumonitis is frequently higher than in clinical trials, with some analyses suggesting rates of 5–10% for PD-1/PD-L1 monotherapy and even higher rates for dual-ICI regimens [32,33,34]. Several factors contribute to this discrepancy. Patients treated in clinical practice frequently have more comorbidities, including chronic obstructive pulmonary disease (COPD), prior thoracic radiation, and smoking-related lung damage, all of which may predispose to pneumonitis or complicate its detection [35,36,37].

In addition, real-world diagnostic practices are heterogeneous. CIP is a diagnosis of exclusion and requires careful evaluation to rule out opportunistic infections, pulmonary embolism, heart failure and tumor progression. In many centers, bronchoscopy is not routinely performed, and radiologic interpretation varies across institutions, leading to variability in case ascertainment. As a result, some cases of CIP may remain unrecognized, while others may be overdiagnosed in patients with infectious pneumonias or TKI-related pulmonary toxicity [32,38]. Grading of severity is similarly inconsistent, particularly for borderline presentations where dyspnea or radiologic changes could stem from multiple etiologies [39,40].

Despite these challenges, real-world evidence highlights an important point: the burden of CIP in RCC may be greater than suggested by clinical trials, particularly in older patients, those with extensive metastatic lung involvement and those treated with combination regimens. Moreover, the outcomes of CIP in routine practice may be worse, as comorbidities and delays in diagnosis can contribute to prolonged hospitalization, need for high-dose corticosteroids or permanent treatment discontinuation [32,38].

The discrepancy between clinical trial and real-world incidence estimates deserves particular consideration. While higher real-world rates may reflect the inclusion of patients with greater pulmonary vulnerability and comorbidity burden, they may also indicate that pneumonitis is under-recognized or under-reported in registration trials because of restrictive eligibility criteria and variability in toxicity attribution. Conversely, the lack of standardized diagnostic algorithms in routine practice may occasionally lead to overestimation, particularly when infectious complications, pulmonary metastases, or TKI-related toxicities mimic CIP. Taken together, these observations suggest that the true burden of pneumonitis in RCC is likely not fully captured by either clinical trials or retrospective real-world analyses, highlighting the need for prospective studies using standardized diagnostic criteria [32,35,36,37,38].

### 2.4. Comparative Perspective with NSCLC

Understanding the epidemiology of CIP in RCC is enriched by comparison with NSCLC, the tumor type in which CIP has been most extensively characterized. In NSCLC, pneumonitis occurs more frequently, with rates as high as 10 to 15 percent for PD-1/PD-L1 monotherapy and even higher for dual-ICI regimens. Several factors explain this increased susceptibility. The presence of underlying lung disease is common in NSCLC, often related to smoking history, emphysema or prior thoracic radiation. In addition, the tumor itself directly involves the pulmonary parenchyma, creating a microenvironment predisposed to inflammation. These baseline vulnerabilities likely amplify the effects of immune activation, leading to higher rates of pneumonitis and earlier symptom onset [10,11,14,41,42].

The earlier and more robust characterization of CIP in NSCLC has resulted in stringent monitoring strategies, including frequent imaging and heightened clinical awareness, which may improve detection [32,43]. By contrast, RCC patients, who often lack pre-existing lung disease, may not undergo the same degree of pulmonary surveillance. Furthermore, CIP is frequently overshadowed by TKI toxicity and metastatic lung involvement [44].

Nevertheless, the experience gained from NSCLC underscores the need for a proactive risk stratification routine, early-treatment imaging in high-risk patients, structured symptom monitoring and rapid evaluation of new respiratory complaints. Adapting these approaches to the RCC population may facilitate earlier diagnosis and better outcomes [39].

## 3. Clinical and Radiological Presentation

The clinical presentation of CIP in patients with renal cell carcinoma is characterized by substantial heterogeneity, reflecting the diverse manifestations of immune-mediated inflammation within the lung parenchyma. Symptoms range from mild and nonspecific to rapidly progressive and life-threatening. This variability contributes to the considerable diagnostic challenge associated with CIP, where pulmonary metastases and treatment-related toxicities may closely mimic the radiological and clinical features of pneumonitis. A systematic understanding of the symptomatology, time to onset, imaging patterns and competing diagnostic considerations is therefore essential for timely recognition and effective management.

### 3.1. Symptomatology

The clinical symptoms of CIP are often subtle in the early stages and can overlap with a wide array of pulmonary and systemic conditions. The most frequently reported complaints include dyspnea, cough and low-grade fever, although some patients present with nonspecific fatigue or chest discomfort. Dyspnea is typically exertional at first and may progress insidiously over days or weeks, eventually occurring at rest in more advanced cases. Cough is often dry and poorly productive, but in some patients it may be accompanied by mild sputum production. Fever, when present, tends to be low grade but can lead clinicians to initially suspect infection, delaying consideration of CIP [13,14,20,45].

In more severe presentations, hypoxia becomes evident and may progress rapidly, leading to respiratory distress that necessitates hospitalization [13,14]. Importantly, a subset of patients remains asymptomatic, particularly when CIP is detected incidentally on routine imaging [32,38]. These cases, typically classified as grade 1 pneumonitis, highlight the role of radiological surveillance in early stage toxicity detection [13]. As combination regimens involving immune checkpoint inhibitors and tyrosine kinase inhibitors become more prevalent, the clinical profile of CIP may be further complicated by overlapping symptoms such as fatigue, dyspnea or cough-induced by the TKI component [29].

### 3.2. Time to Onset Across Different Therapeutic Regimens

The time to onset of CIP varies considerably according to the type of immunotherapy regimen administered. Monotherapy with PD-1 or PD-L1 inhibitors (such as nivolumab or pembrolizumab) is associated with a broad range of onset times, typically from several weeks to many months after initiation, with median onset reported between 2 and 5 months, depending on the individual immune response. This relatively gradual emergence aligns with the mechanism of action of PD-1 blockade, which modulates existing T cell responses rather than inducing rapid immune priming [13,24,32,39,46].

Dual immune checkpoint inhibition, most commonly involving nivolumab plus ipilimumab, is associated with higher incidence and earlier onset of CIP. In pivotal trials and meta-analyses, most grade 3–4 immune-related adverse events—including pneumonitis—occurred within the first 4–6 months of treatment. The earlier onset reflects the combined immune activating effects of PD 1 and CTLA-4 inhibition, which together enhance both T cell priming and effector function, thereby increasing the likelihood of immune-mediated inflammation in the lung parenchyma [13,47].

In contrast, patients treated with combinations of immune checkpoint inhibitors and tyrosine kinase inhibitors tend to exhibit a broader range of onset times. CIP in this setting may occur either early, particularly when the ICI component induces a brisk immune response, or later, after several months of combination therapy. The variability is partly attributable to the overlapping toxicities induced by VEGF-targeted tyrosine kinase inhibitors, which can cause nonspecific pulmonary symptoms or radiological changes that obscure the early inflammatory phase of CIP. As a result, CIP in ICI–TKI regimens may remain undetected until significant radiological progression or symptomatic decline prompts further evaluation [29,48,49].

### 3.3. Radiologic Patterns on Computed Tomography

Chest computed tomography is essential in the diagnostic evaluation of CIP, revealing characteristic patterns of inflammation that provide clues regarding severity and underlying pathophysiology. Although no single radiological finding is pathognomonic for CIP, several recurrent patterns have been recognized across studies and clinical experience.

The most commonly encountered pattern is organizing pneumonia. This is characterized by patchy or confluent areas of consolidation that often exhibit a peripheral or peribronchial distribution. The lesions may be unilateral or bilateral and frequently coexist with ground-glass opacities. The organizing pneumonia pattern is associated with a relatively favorable prognosis and a good response to corticosteroids, though progression can occur in the absence of timely intervention [13,32,38,50,51,52,53].

Nonspecific interstitial pneumonia is another frequent pattern observed in CIP. It manifests as diffuse or subpleural ground-glass opacities with or without fine reticulation. The distribution is typically bilateral and symmetric. Although this pattern is less common than organizing pneumonia, it may be associated with more diffuse pulmonary involvement and, occasionally, impaired gas exchange [51,52,54].

Ground-glass opacities, either isolated or superimposed upon other patterns, are a hallmark of early inflammatory changes in CIP. They reflect alveolar and interstitial inflammation and can be reversible with prompt management. However, ground-glass opacities alone are nonspecific and may be seen in infections, edema or hemorrhage, underscoring the need for a thorough differential diagnosis [51,52,54].

A hypersensitivity pneumonitis-like pattern is reported less frequently but remains an important diagnostic consideration. It usually consists of diffuse or patchy ground-glass opacities accompanied by centrilobular nodules or subtle mosaic attenuation. This pattern reflects a more generalized immune reaction within the lung and may present with more pronounced respiratory symptoms [51,52].

The radiologic diversity of CIP is emphasized in the Fleischner Society position paper, which notes that a single drug can be associated with multiple CT patterns and that distinguishing CIP from pulmonary metastases or treatment-related changes requires expert interpretation and often serial imaging. Longitudinal CT follow-up is recommended to assess pneumonia activity and resolution, particularly in complex cases or when radiologic findings overlap with other etiologies [54].

### 3.4. Diagnostic Pitfalls Unique to Renal Cell Carcinoma

One of the most significant pitfalls is the presence of pulmonary metastases, which are common in advanced renal cell carcinoma and may exhibit imaging characteristics that closely resemble CIP. Metastatic nodules may develop surrounding ground-glass changes due to hemorrhage, edema or treatment response, mimicking the organizing pneumonia or nonspecific interstitial pneumonia patterns associated with CIP. Conversely, CIP-related inflammation may obscure or distort existing metastatic lesions, making differentiation even more difficult [11,14,42].

Another major source of diagnostic confusion is the heightened risk of pulmonary infections in patients receiving VEGF-targeted tyrosine kinase inhibitors, corticosteroids or prior immunosuppressive therapies. A meta-analysis found that VEGFR-TKI significantly increases the risk of severe and fatal infections, including pneumonia and sepsis, by impairing mucosal integrity or altering immune cell trafficking [55]. Furthermore, early use of corticosteroids for the management of other immune-related adverse events can mask the initial symptoms of infection or CIP, delaying accurate diagnosis. Opportunistic pathogens, including pneumocystis jirovecii, atypical bacteria and respiratory viruses, may produce radiologic patterns that overlap significantly with CIP, necessitating bronchoscopy and microbiological analysis in ambiguous cases [13,38,42].

Cardiopulmonary comorbidities also play an important role in the differential diagnosis. Thromboembolic disease, which occurs with increased frequency in renal cell carcinoma due to tumor-induced hypercoagulability and treatment-related factors, can present with acute dyspnea and hypoxia that closely resemble severe CIP. Computed tomography pulmonary angiography is often required to distinguish between these conditions [45,53]. Heart failure, either pre-existing or induced by VEGF-targeted therapies, can lead to pulmonary edema manifesting as bilateral ground-glass opacities, further complicating the diagnostic landscape. The American Heart Association and American College of Cardiology note that VEGFR-TKIs (e.g., sunitinib, pazopanib) are associated with left ventricular dysfunction and heart failure [50,56].

In summary, the clinical and radiological presentation of CIP is multifaceted and often confounded by competing diagnoses. The interplay between immune-related toxicity, metastatic disease and treatment-related pulmonary effects necessitates a high index of suspicion and a multidisciplinary approach to evaluation. Early recognition and differentiation from alternative etiologies are critical to ensuring appropriate management and optimizing patient outcomes.

## 4. Pathophysiology and Mechanisms

**Figure 1:**
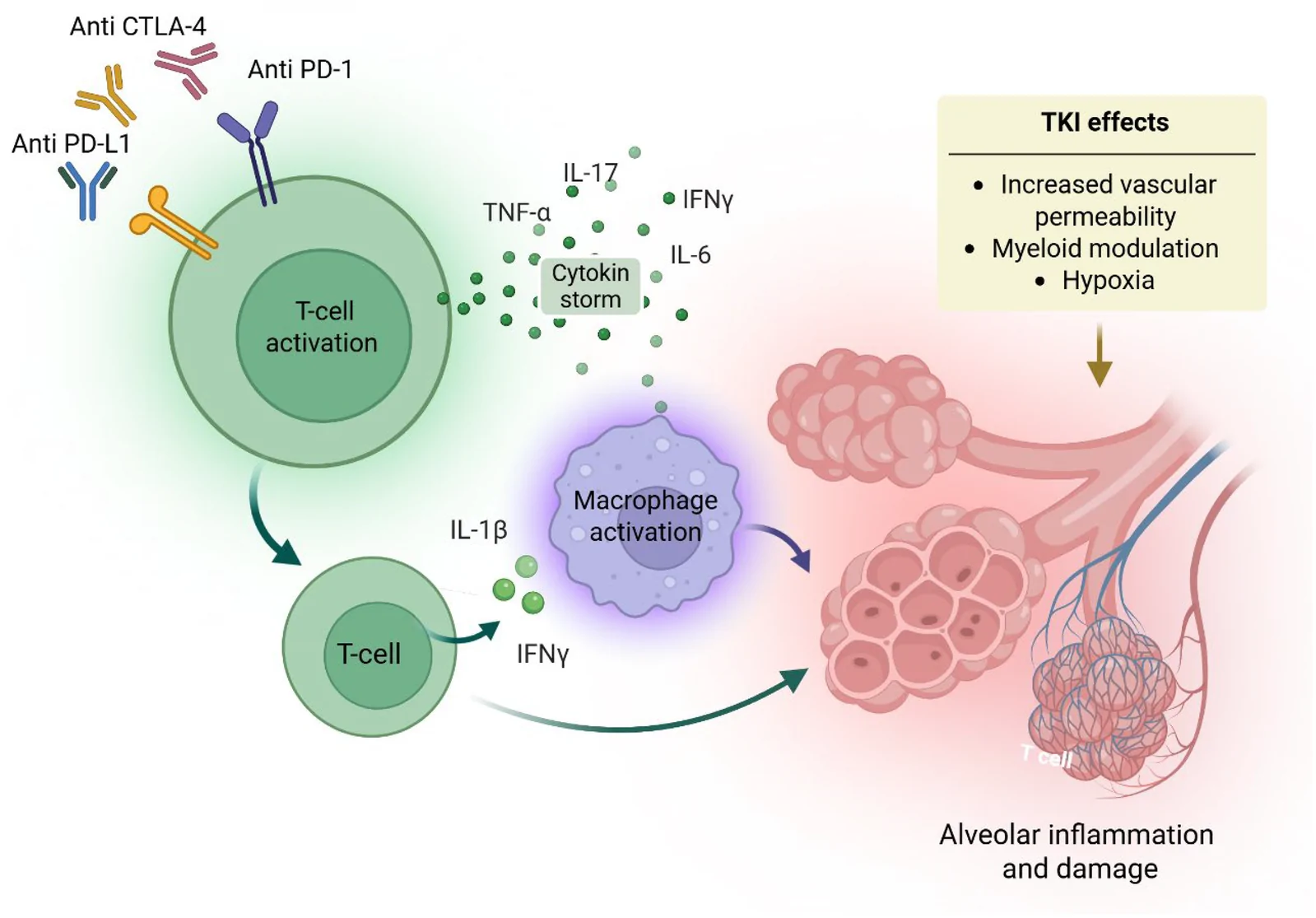
Proposed mechanisms of immune checkpoint inhibitor-related pneumonitis. Schematic representation of the mechanisms underlying immune checkpoint inhibitor-related pneumonitis (CIP). Immune checkpoint blockade (anti-PD-1, anti-PD-L1, and anti-CTLA-4) promotes T cell activation and the release of pro-inflammatory cytokines (including IFN-γ, IL-6, TNF-α, and IL-17), leading to macrophage activation and immune-mediated alveolar injury. Tyrosine kinase inhibitors (TKIs) may further contribute through vascular permeability changes, myeloid cell modulation, and hypoxia, amplifying pulmonary inflammation and resulting in alveolar damage.

The pathophysiology of CIP reflects a complex interplay between immune activation, innate and adaptive inflammatory cascades and the unique tumor and host characteristics that shape pulmonary vulnerability. Although the precise mechanisms remain incompletely elucidated, accumulating preclinical and clinical evidence suggests that pneumonitis arises from dysregulated immune responses targeting the alveolar and interstitial compartments of the lung (Figure 1). In renal cell carcinoma, additional layers of complexity emerge from the widespread use of combination regimens involving TKI targeting vascular endothelial growth factor signaling. These agents exert independent and synergistic effects on vascular permeability, immune cell trafficking and myeloid cell biology, potentially modifying the susceptibility and phenotype of pneumonitis in this specific patient population [55,56,57,58,59,60,61].

### 4.1. Immune-Mediated Alveolar and Interstitial Injury

The lung is a highly immune-active organ, constantly exposed to environmental antigens and equipped with a dense network of resident immune cells, including alveolar macrophages, dendritic cells and tissue resident memory T lymphocytes. Under physiological conditions, these cells maintain a tightly regulated balance between immune surveillance and tolerance. ICI disrupts this equilibrium by blocking inhibitory pathways that normally restrain inappropriate or excessive immune activation, leading to direct cytotoxic injury of alveolar epithelial cells and interstitial tissues [12,43,57,58,59].

Histopathologically, the most common pattern demonstrated by analyses of lung biopsy specimens from patients with immune checkpoint inhibitor-related pneumonitis is organizing pneumonia, characterized by intra-alveolar fibroblastic plugs and chronic interstitial inflammation. Other patterns include non-specific interstitial pneumonia, hypersensitivity pneumonitis, acute fibrinous pneumonitis, and diffuse alveolar damage, the latter two associated with several or fatal cases. These patterns indicate a combination of T cell-mediated cytotoxicity and innate immune activation, resulting in compromised alveolar integrity, impaired gas exchange and accumulation of inflammatory exudates. Foamy macrophages, pneumocyte vacuolization, and rare eosinophilis can also be observed. The diversity of pathological changes likely accounts for the wide spectrum of radiological patterns observed on computed tomography, ranging from ground-glass opacities to consolidations and reticulations [38,51,52,60].

### 4.2. T Cell Infiltration, Macrophage Activation and Cytokine Dysregulation

The underlying immunological mechanisms of CIP involve excessive activation and expansion of cytotoxic T lymphocytes and helper T cells, downregulation of regulatory T cells, and overproduction of pro-inflammatory cytokines such as IFN-y and IL-17A. There is also dysregulation of innate immune cells, including increased inflammatory monocytes, dendritic cells, and M1-polarized macrophages, with impaired differentiation toward anti-inflammatory macrophages. Single-cell analyses of bronchoalveolar lavage fluid in CIP demonstrate accumulation of CXCL13+ T cells with hyperexpanded TCR clonotypes and hyperinflammatory CXCL9+ monocytes, with activation of CXCL9/10/11-CXCR3, FASLG-FAS and IFNGR1/2-IFNG signaling pathways, and evidence of B cell activation and macrophage pyroptosis [62,63,64,65,66,67].

Effector T cells, especially cytotoxic CD8-positive T cells, are central to the pathogenesis of CIP. Unchecked activation of these cells leads to direct epithelial injury as well as the release of inflammatory mediators that recruit additional immune cells and amplify local inflammation. Increased infiltration of CD4-positive helper T cells, including Th1 and Th17.1 subsets, has also been documented, indicating broad activation of adaptive immunity and further cytokine production [62,63,64].

Activated T cells in CIP produce cytokines such as interferon gamma and tumor necrosis factor alpha, which contribute to the amplification of inflammation and disruption of alveolar homeostasis [58,59,61,62,63,64]. Macrophages, particularly alveolar macrophages, adopt a proinflammatory phenotype in the presence of heightened T cell activation, secreting IL-6, IL-1B, and other mediators that perpetuate tissue injury. IL6 is implicated in immune-related toxicities and promotes vascular leakage, fibroblast activation, and systemic inflammation. The combined effects of these cytokines create a feedback loop that sustains the inflammatory process even after the initial immune trigger, and the degree of cytokine dysregulation may explain why some cases of pneumonitis progress rapidly and require high-dose corticosteroids or additional immunosuppressive therapy [65,66,67].

**Figure 2:**
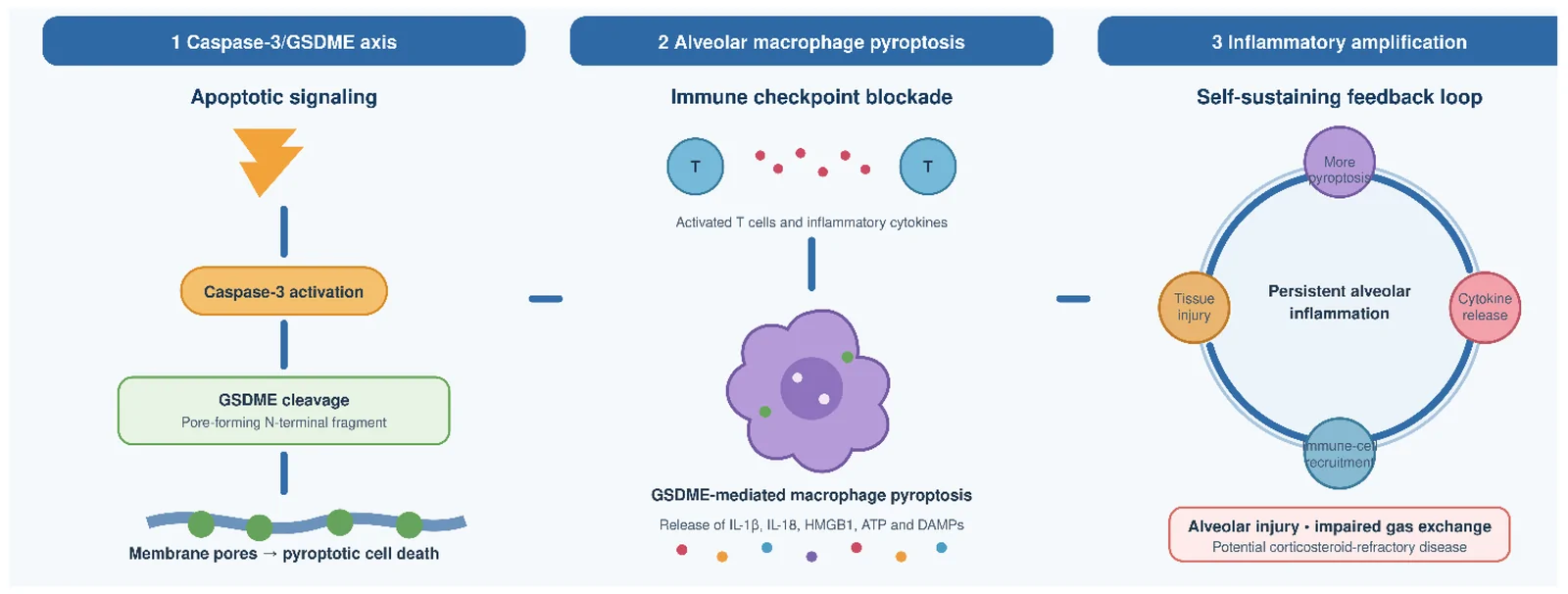
GSDME-mediated pyroptosis and macrophage-driven lung injury in immune checkpoint inhibitor-related pneumonitis (CIP). The figure summarizes a proposed mechanism underlying severe and refractory CIP. (1) Activation of caspase-3 downstream of apoptotic signaling induces cleavage of gasdermin E (GSDME), releasing its N-terminal pore-forming fragment. GSDME-mediated membrane pore formation converts apoptosis into inflammatory pyroptotic cell death, leading to cell swelling, membrane rupture, and release of pro-inflammatory mediators and damage-associated molecular patterns (DAMPs). (2) During immune checkpoint blockade, activated T cells and inflammatory cytokines promote alveolar macrophage activation. Increased expression and cleavage of GSDME in macrophages trigger pyroptosis, amplifying pulmonary inflammation through the release of IL-1β, IL-18, HMGB1, ATP, and other inflammatory mediators. This process contributes to alveolar injury, inflammatory cell infiltration, and impaired lung function characteristic of CIP. (3) Pyroptotic macrophages establish a self-sustaining inflammatory feedback loop by recruiting and activating additional macrophages and immune cells. Persistent cytokine production enhances tissue injury, promotes further macrophage pyroptosis, and may drive corticosteroid-refractory disease. Potential therapeutic strategies include inhibition of GSDME activation, blockade of inflammatory cytokines, and modulation of macrophage-mediated immune responses to interrupt this pathogenic cycle.

Recent evidence suggests that macrophage pyroptosis may represent a key mechanism underlying severe and steroid-refractory immune checkpoint inhibitor-related pneumonitis. Pyroptosis is a highly inflammatory form of programmed cell death characterized by gasdermin-mediated membrane pore formation and release of pro-inflammatory cytokines. Experimental studies have demonstrated that GSDME-mediated macrophage pyroptosis may amplify pulmonary inflammation through the release of IL-1β and other inflammatory mediators, promoting a self-sustaining inflammatory loop within the lung microenvironment (Figure 2). This mechanism may contribute to persistent alveolar damage and explain, at least in part, the resistance observed in some cases despite high-dose corticosteroid treatment. Although current evidence remains largely preclinical, macrophage pyroptosis represents a promising area for future translational research and potential therapeutic intervention [60,63,64].

### 4.3. Potential Genetic, Microbiome and Autoimmune-Related Predispositions

Although immune checkpoint inhibitor treatment is the primary driver of pneumonitis, host-specific factors likely influence susceptibility. Genetic predisposition is an area of growing research, with emerging data suggesting that certain human leukocyte antigen haplotypes (HLA), such as HLA-B35 and DRB111, are associated with increased risk of CIP, and that homozygosity of HLA-I loci and specific HLA supertypes (e.g., HLA-A03) influence susceptibility to immune-related adverse events, including pneumonitis. Genomic-wide association studies have identified single nucleotide polymorphisms (SNPs) such as rs1929211786, which are associated with increased risk of pneumonitis in patients treated with PD-1/PD-L1 inhibitors, and immunogenetic variants in genes regulating immune tolerance and cytokine signaling (e.g., MAPK1, PTPRC, ADAD1, IL6) also modulate risk [68,69,70,71].

The lung microbiome represents another potential contributor. Alterations in microbial diversity and composition, such as enrichment of Prevotella Melanigonica or increased abundance of candida, vibrio, and Halomomas, are associated with exaggerated immune response and higher risk of pneumonitis. These microbial changes correlate with host immune-inflammatory markers and may prime the lung for immune checkpoint inhibitor-related toxicity [68,69,70].

There is increased risk of pneumonitis in patients with a history of autoimmune disease, especially those with pulmonary involvement, or in those with subclinical autoimmunity or circulating autoantibodies. Pre-existing autoimmune disease is an independent risk factor for immune-related adverse events, including pneumonitis, with a higher odds of occurrence and earlier onset. The presence of auto-reactive antibodies, such as those against AChR3, CXCL10, BAFF, and cytokeratin 19, is associated with increased risk of CIP, and these antibodies may serve as predictive biomarkers for susceptibility [72,73,74,75,76].

### 4.4. Added Complexity in Renal Cell Carcinoma Treated with Immune Checkpoint Inhibitors and Tyrosine Kinase Inhibitors

The pathophysiology of pneumonitis in renal cell carcinoma must be interpreted in the context of modern treatment paradigms, which increasingly rely on combinations of immune checkpoint inhibitors and tyrosine kinase inhibitors targeting vascular endothelial growth factor pathways. These combinations create a unique biological environment in which immune activation is superimposed upon profound changes in vascular biology and myeloid cell function.

Inhibition of VEGF signaling alters endothelial barrier integrity and increases vascular permeability within the lung, facilitating extravasation of immune cells, cytokines, and plasma proteins into the interstitial and alveolar spaces, which can amplify lung inflammation initiated by ICIS. VEGF is a potent inducer of vascular permeability, and its blockade can paradoxically increase pulmonary edema or hemorrhage, which may radiologically mimic pneumonitis and complicate interpretation [28,38,46].

TKIs exert additional effects on myeloid cells, including modulation of dendritic cell maturation, inhibition of immunosuppressive myeloid-derived suppressor cells (MDSCs) and alteration of alveolar macrophage function. These changes may enhance antitumor immunity but also create conditions favorable for exaggerated pulmonary inflammation [59,71,72,73].

Anti-angiogenic therapy can induce tissue hypoxia, which is a potent driver of inflammation signaling and may lower the threshold for immune-mediated alveolar injury. Hypoxia activates hypoxia-inducible factors (HIFs), which regulate a wide variety of genes involved in inflammation, immune cell recruitment, and vascular permeability. Hypoxia-induced inflammation is clinically relevant, as it promotes vascular leakage, accumulation of inflammatory cells, and elevated cytokine levels in the lung [29,32,48,50,52,53].

The combination of ICIS with TKIs introduces multiple synergistic mechanisms that could increase the risk or modify the phenotype of pneumonitis [59,71]. Meta-analyses and systematic reviews confirm that ICI–TKI combinations are associated with a high incidence of grade 3–5 toxicity (approximately 56%) with overlapping pulmonary effects from both agents complicating diagnosis and management [38,74].

### 4.5. Differences in Mechanism Compared with Non-Small Cell Lung Cancer (NSCLC)

Although the immunopathology of pneumonitis shares core features across tumor types, the mechanisms in renal cell carcinoma differ in important ways from those in NSCLC. Differences between these tumors are strongly influenced by the impact of pre-existing structural lung abnormalities (e.g., emphysema), tumor microenvironment, prior thoracic radiation, and the role of systemic immune activation and TKI in RCC [37,77]. These conditions increase pulmonary fragility and reduce the capacity of the lung to tolerate immune-mediated inflammation.

Pre-existing structural lung abnormalities such as interstitial lung disease (ILD), interstitial lung abnormalities (ILA), and prior radiation pneumonitis are major risk factors for CIP in non-small cell lung cancer, with odds ratios for pneumonitis ranging from 3.6 to 6.6 in patients with these findings [76]. These conditions are less common in renal cell carcinoma, where the baseline lung parenchyma is typically normal, resulting in lower intrinsic risk of pneumonitis from ICI [29,32,38].

Prior thoracic radiation synergistically increases the risk and severity of CIP in NSCLC by causing microvascular damage, fibroblast activation and chronic low-grade inflammation. It presents a distinct radiologic morphology, tending to be unilateral with sharp borders. In RCC, thoracic radiation is rarely used, so this risk factor is less relevant [78,79,80].

The tumor microenvironment in RCC is characterized by chronic inflammation, hypoxia, abnormal vasculature, and immune exclusion, with prominent roles for angiogenesis, myeloid-derived suppressor cells, and carcinoma-associated fibroblasts [59,71,77]. This contrasts with NSCLC, where the microenvironment is shaped by direct tumor involvement of the lung, smoking-related damage, and prior radiation, leading to increased susceptibility to immune-related injury [35,36,76].

Systemic immune activation in RCC is modulated by the unique tumor microenvironment, with variable infiltration of cytotoxic and immunosuppressive immune cells, and is further influenced by combining therapy with tyrosine kinase inhibitors targeting VEGF pathways [59,71,77]. These mechanisms are less prominent in NSCLC, where TKI are not standard in combination with ICIs [29,74]. Therefore, pneumonitis in renal cell carcinoma may rely more heavily on systemic factors than on local pulmonary vulnerability [32,59].

These distinctions underscore why data derived from NSCLC, although informative, cannot be directly extrapolated to RCC and why disease-specific research on pneumonitis mechanisms and risk factors remains critically needed [43,81,82,83].

## 5. Risk Factors in RCC

Before discussing individual risk factors, it is important to acknowledge that the evidence supporting susceptibility to CIP varies considerably across tumor types. Current knowledge can be broadly categorized into three evidence tiers: (i) RCC-specific evidence derived from clinical trials and observational studies conducted in renal cell carcinoma populations; (ii) mixed solid-tumor evidence, including cohorts in which RCC patients represented a subset of the study population; and (iii) NSCLC-derived evidence, where risk factors have been more extensively characterized because of the higher incidence of pneumonitis and the larger body of available data. Throughout this section, we distinguish these levels of evidence to avoid overgeneralization and to highlight areas where RCC-specific data remain limited [32,38].

Understanding the risk factors predisposing patients with RCC to CIP is essential for optimizing patient selection, designing surveillance strategies and improving outcomes through early recognition. Although the overall incidence of pneumonitis in RCC is lower than in NSCLC, a number of clinical, biological and treatment-related elements may increase susceptibility in specific subgroups. Unlike NSCLC, where underlying lung pathology and tumor location strongly influence the risk of pneumonitis, the determinants of susceptibility in renal cell carcinoma are shaped by systemic factors, comorbid conditions and the unique therapeutic landscape of this disease. These considerations highlight the importance of a disease-specific approach to risk stratification [29,32,35,38,59].

### 5.1. Baseline Comorbidities and Pulmonary Vulnerability

Baseline pulmonary comorbidities represent a central risk factor for pneumonitis in patients receiving ICI. COPD is a well-established risk factor for CIP. A meta-analysis found that patients with pre-existing COPD had a significantly increased risk of CIP (OR 1.54–1.97, 95%, CI 1.24–2.76), with pooled incidences of any grade and grade ≥ 3 CIP of 20% and 6%, respectively, in patients with COPD. These data are mostly from lung cancer cohorts and extrapolated to RCC. The NCCN guidelines recommend considering pulmonary function tests with diffusion capacity for patients at high risk, including those with COPD. Even mild situations can predispose to rapid symptom escalation once alveolar injury occurs, as these patients have chronic airway inflammation, impaired mucociliary clearance, and reduced pulmonary reserve [51,52].

Pre-existing ILD, whether idiopathic or secondary to autoimmune disorders, is a major risk factor for CIP across all tumor types, with meta-analyses reporting OR of 3.2–5.8 for any-grade CIP across ICI-treated populations and 2.9 for grade ≥ 3 CIP in patients with pre-existing ILD. These patients have higher recurrence rates (16.4% vs. 8–9%) and higher all-cause mortality (41.1%) when CIP develops. The NCCN guidelines recommend heightened vigilance and consideration of pulmonary function tests in patients with ILD on imaging. This condition may also trigger acute exacerbation characterized by diffuse alveolar damage and rapid respiratory decline [32,38,50,53].

Prior thoracic radiation therapy represents another potential contributor to increased risk, though its less common in RCC than NSCLC, with meta-analyses reporting an OR of 2.07–4.1 for CIP development. Radiation induces long-term structural and microvascular changes, including fibrosis, endothelial dysfunction and chronic low-grade inflammation, which create a sensitized pulmonary environment where additional immune activation may produce exaggerated injury. Thoracic irradiation is less frequently used in RCC, but patients may have received radiation for metastatic lesions or other primary cancers earlier in life [14,42,43,55].

Smoking history also appears to increase the risk of CIP, with meta-analyses reporting an OR of 1.39 (95% CI 1.14–1.71) among smokers. Smoking causes oxidative injury, epithelial dysfunction, and chronic airway inflammation, all of which impair the lung’s ability to respond appropriately to immune challenges. Smoking history often correlates with the presence of emphysema, bronchial thickening, or subclinical interstitial abnormalities that may not be apparent on routine imaging but nevertheless influence risk. The cumulative effect of smoking-induced damage and ICI may shift the balance toward harmful rather than therapeutic immune activation [45,56].

Importantly, most quantitative estimates reported for COPD, ILD, smoking history and prior thoracic radiation originate from NSCLC or mixed solid-tumor cohorts and should therefore be interpreted as indirect evidence when applied to RCC populations [32,35,36].

### 5.2. Treatment-Related Factors and Regimen-Specific Risks

Treatment-related factors are among the most important determinants of pneumonitis risk in renal cell carcinoma, particularly in the era of combination regimens involving immune checkpoint inhibitors and tyrosine kinase inhibitors. The incidence of pneumonitis varies considerably according to the therapeutic strategy, reflecting differences in immune activation, off-target drug effects and cumulative toxicity. This is supported by meta-analyses and network meta-analyses showing that the incidence and risk of pneumonitis differ significantly between ICI monotherapy, dual ICI combinations, and ICI–TKI combinations [29,84].

Dual checkpoint blockade (nivolumab plus ipilimumab) is consistently associated with a higher incidence of pneumonitis than monotherapy with PD-1 or PD-L1 inhibitors. A meta-analysis found that patients receiving combination therapy were significantly more likely to experience pneumonitis, both any-grade (OR 2.04–2.13, 95% CI 1.69–2.63, *p* < 0.001) and grade 3 or higher pneumonitis (OR 2.78–2.86, 95% CI 1.61–4.76, *p* < 0.001). This increased risk is thought to result from the simultaneous activation of T cell priming through CTLA-4 inhibition and enhancement of effector function through PD-1 inhibition. The compounded immune stimulation may overwhelm regulatory mechanisms within the lung, leading to more robust inflammatory responses [20,26,85].

The risk associated with combinations of ICI and TKI appears to be more nuanced. While these regimens typically demonstrate lower reported incidences of pneumonitis in clinical trials, real-world data suggest that diagnostic challenges may obscure the true risk. TKI targeting VEGF pathways can cause dyspnea, cough and radiologic changes independent of immune mechanisms. These effects include increased vascular permeability, transient interstitial edema and alterations in immune cell trafficking, all of which can complicate the detection of pneumonitis. Whether TKIs synergistically amplify immune-related pulmonary injury remains unclear. Some mechanistic studies suggest that vascular endothelial growth factor inhibition may enhance T cell infiltration by modifying endothelial barrier function, potentially lowering the threshold for pneumonitis during immune checkpoint inhibitor exposure. Conversely, other studies propose that tyrosine kinase inhibitors may have immunomodulatory effects that reduce inflammation in certain contexts. This apparent duality contributes to ongoing uncertainty regarding the true risk profile of these regimens [28,86,87,88,89].

Cumulative toxicity is another important hypothesis for treatment-related factors that may influence the risk of pneumonitis, particularly in later line settings. Patients who receive multiple sequential therapies often have decreased physiological reserve, immune dysregulation induced by prior treatments and increased exposure to corticosteroids or immunosuppressive drugs. These elements may render the lung more vulnerable to subsequent inflammatory injury and prolong recovery once pneumonitis develops. Additionally, late-line tyrosine kinase inhibitors may contribute to chronic endothelial dysfunction, further compounding susceptibility [90,91,92].

In contrast, evidence regarding treatment-related risk is more directly supported by RCC-specific clinical trials evaluating dual-ICI and ICI–TKI combinations.

### 5.3. Patient-Specific Features Distinguishing RCC from NSCLC

Patients with RCC rarely have intraparenchymal tumors that directly disrupt or infiltrate the lung architecture, whereas patients with NSCLC often harbor large primary tumors or multiple parenchymal metastases that create a proinflammatory microenvironment. Metastatic tumors from RCC tend to show more expansive growth in the lung rather than infiltrative growth, with adjacent lung retraction more typical of primary lung cancers. Pulmonary metastases from RCC can grow via distinct patterns (destructive, alveolar, interstitial), with the alveolar and destructive patterns seen in primary lung cancers. Tumor-induced inflammation, mechanical obstruction, and tumor necrosis within the lung are common drivers of pulmonary fragility in NSCLC, predisposing to pneumonitis when ICIs are administered [68,69,70,71].

Renal cell carcinoma patients often receive sequential TKI before being treated with ICI, particularly in earlier therapeutic eras before combination regimens became standard. Prolonged exposure to TKIs can alter vascular integrity, immune cell dynamics, and tissue oxygenation, potentially modifying the baseline pulmonary environment. In contrast, patients with NSCLC often receive chemotherapy or radiation as first-line therapy, each with distinct pulmonary effects that influence susceptibility to pneumonitis [72,73,74,75,76,93].

Moreover, the timing of ICI introduction differs significantly between the two tumor types. In renal cell carcinoma, ICIs are often initiated when patients have relatively preserved lung function and limited pulmonary symptoms, whereas in NSCLC, ICIs are frequently introduced in the presence of pre-existing dyspnea, chronic hypoxia, or radiation-induced fibrosis, all of which heighten vulnerability [10,35,37,77]. The incidence of pneumonitis is higher and occurs earlier in NSCLC than in non-lung cancers (RCC, melanoma), with a median of 78 days vs. 186 days in non-lung cancers [24,53].

Finally, biological differences between the two cancers may influence immune-related toxicities. RCC is characterized by a highly vascular microenvironment and a prominent role for myeloid-derived suppressor cells [79,81,82], whereas NSCLC arises in a structurally altered and immunologically primed organ [10,37,54,77]. These distinctions shape the immune context in which checkpoint inhibition occurs and likely contribute to differences in CIP risk and phenotype [32,43,81,82].

These observations further support the need to interpret NSCLC-derived risk models cautiously when evaluating RCC patients.

## 6. Diagnosis and Management

The diagnosis and management of CIP in RCC require a structured and multidisciplinary approach. Early recognition is essential because pneumonitis may progress rapidly from mild subclinical disease to severe respiratory compromise, and delayed treatment is associated with worse outcomes. However, the diagnosis is frequently challenging, especially in RCC where pulmonary metastases, infectious complications and toxicities associated with TKIs often mimic or obscure the presentation of pneumonitis. To address these complexities, clinicians must integrate clinical, radiologic and laboratory findings and adhere to evidence-based management principles derived from guidelines issued by the American Society of Clinical Oncology (ASCO), the European Society for Medical Oncology (ESMO) and the Society for Immunotherapy of Cancer (SITC) (Table 2).

### 6.1. Diagnostic Approach

HRCT plays a central role in the diagnostic evaluation of suspected pneumonitis. The Fleischner Society position paper states that imaging features of drug-related pneumonitis should be assessed using a radiologic pattern approach, with CT patterns reflecting acute (diffuse alveolar damage), subacute (organizing pneumonia and hypersensitivity pneumonitis), and chronic (nonspecific interstitial pneumonia) interstitial disease. The NCCN guidelines recommend chest CT with contrast to evaluate for pneumonitis and to rule out other etiologies. Serial imaging is useful to assess evolution over time and response to treatment [38,51,60].

Bronchoscopy with bronchoalveolar lavage (BAL) is a key tool in the evaluation of suspected pneumonitis, particularly when the radiologic findings are equivocal or when infectious etiologies cannot be confidently excluded. The NCCN guidelines recommend early bronchoscopy with BAL for grade ≥ 2 pneumonitis to rule out infection, malignant lung infiltration, or other steroid-responsive ILD [94,95,96,97]. It allows direct sampling of airway secretions and enables microbiologic cultures, PCR assays for atypical pathogens, and cytologic assessment. Infections such as Pneumocystis jirovecii pneumonia, fungal organisms, or viral pathogens may produce radiologic abnormalities that resemble pneumonitis, especially in patients who have recently received systemic corticosteroids or TKIs. Furthermore, bronchoscopy may help differentiate pneumonitis from pulmonary hemorrhage, a complication that is occasionally observed in renal cell carcinoma due to tumor vascularity or vascular endothelial growth factor pathway inhibition.

A comprehensive differential diagnosis is essential, particularly for patients receiving combination regimens that include tyrosine kinase inhibitors. TKI toxicity can result in pulmonary edema, interstitial infiltrates or pleural effusions that closely resemble immune-mediated pneumonitis. The Fleischner Society position paper provides diagnostic criteria for differentiating drug-related pneumonitis, diffuse alveolar hemorrhage, pulmonary edema, radiation pneumonitis, and pulmonary lymphangitic carcinomatosis. Other important conditions that must be ruled out include pulmonary embolism, heart failure exacerbation, and progression of metastatic disease. Laboratory tests such as arterial blood gas analysis, biomarkers of cardiac stress, inflammatory markers, and complete blood counts may provide supportive evidence but are rarely diagnostic in isolation. Ultimately, the diagnosis relies on clinical judgment informed by a combination of imaging, bronchoscopy, laboratory data, and response to initial therapy or response to temporary interruption of the tyrosine kinase inhibitor [39,98].

### 6.2. Grading and Management Strategies

Current management of CIP is guided by severity grading according to standardized criteria from ASCO, ESMO, and SITC. Severity is graded using the Common Terminology Criteria for Adverse Events (CTCAE):

- Grade 1 (asymptomatic, radiographic findings only): Management should be individualized. In selected patients, ICI therapy may be continued with close clinical and radiologic monitoring, whereas temporary treatment interruption may be considered in cases with more extensive radiographic involvement or diagnostic uncertainty. Repeat imaging within 2–4 weeks is recommended. Corticosteroids are not recommended unless symptoms develop. Repeat imaging in 3–4 weeks is advised. If radiologic abnormalities persist or progress, escalation of treatment may be warranted.
- Grade 2 (mild to moderate symptoms, limiting instrumental ADLs): Hold ICI therapy and initiate oral corticosteroids (prednisone 1–2 mg/kg/day or equivalent). Bronchoscopy with Bal should be considered to exclude infection. Taper steroids over at least 4–6 weeks. Resume ICI only if symptoms resolve to ≤grade 1 and after steroid taper. Follow-up imaging is recommended within several weeks to evaluate response.
- Grade 3 (severe symptoms, limiting self-care ADLs, hypoxia): Permanently discontinue ICI therapy. Hospitalize the patient and initiate intravenous corticosteroids (methylprednisone 1–2 mg/kg/day). If no improvement within 48 h, add additional immunosuppressive agents such as mycophenolate mofetil, infliximab, or IVIG once infection has been excluded. Bronchoscopy is recommended to exclude infections.
- Grade 4 (life-threatening respiratory compromise): Permanently discontinue ICI therapy. Admit to intensive care, provide high-dose intravenous corticosteroids (methylprednisone 2–4 mg/kg/day), and consider additional immunosuppression as above. Supportive care including oxygen and ventilator support is required.

All societies emphasize multidisciplinary management, early recognition, and rigorous exclusion of infectious etiologies before initiating additional immunosuppression.

### 6.3. Corticosteroid Regimens, Tapering and Escalation

Corticosteroids remain the cornerstone of treatment for pneumonitis, and their timely administration is associated with favorable outcomes. The duration of corticosteroid therapy depends on the severity of pneumonitis at presentation and the patient’s response. The standard tapering duration for grade ≥ 2 pneumonitis is at least 4–6 weeks but longer tapers ≥ 12 weeks may be required for chronic or refractory cases. Tapering should be individualized based on clinical and radiological response, as well as risk of flare, which can occur even after discontinuation of ICI therapy.

Patients should be monitored for steroid-related toxicities, including hyperglycemia, infection, osteoporosis, myopathy and psychiatric effects. Prophylaxis for opportunistic infections (e.g., pneumocystis jirovecii pneumonia) should be considered for those on prolonged or high-dose steroids. Regular clinical, laboratory, and imaging follow-up is essential to assess for resolution of pneumonitis and to detect complications.

Some patients fail to respond adequately to high-dose corticosteroids or deteriorate despite initial improvement. These cases are categorized as steroid-refractory pneumonitis and require escalation to second-line immunosuppressive therapies. Before administering additional immunosuppression, clinicians must rigorously exclude infections, because intensified immunosuppressive therapy can exacerbate underlying pathogens [99,100,101,102,103,104].

### 6.4. Second-Line Immunosuppressants for Steroid–Refractory Pneumonitis

If there is no clinical improvement after 48–72 h of corticosteroid therapy, the pneumonitis is considered steroid-refractory. Additional immunosuppressive agents should be considered.

Infliximab (TNF-alpha inhibitor) is frequently used, with registry and cohort data showing potential for earlier symptom improvement compared to mycophenolate mofetil or IVIG. However, mortality rates are high in steroid-refractory pneumonitis, especially in those treated with infliximab (up to 100% in some series) and infectious complications are common. Infliximab is more established for steroid-refractory colitis, and its use in pneumonitis should be reserved for severe, rapidly progressive cases after thorough exclusion of infection [105,106].

Mycophenolate mofetil is considered a preferred agent for steroid-refractory pneumonitis based on evidence from systematic reviews and case series, with clinical improvement reported in a subset of patients. It offers a complementary approach through inhibition of lymphocyte proliferation and is generally well tolerated, but monitoring for cytopenias and infection is required.

Intravenous immunoglobulins (IVIG) have demonstrated benefit in severe, steroid-refractory cases through modulation of Fc receptor signaling and attenuation of antibody-mediated inflammation. It may be considered in patients with contraindications to cytotoxic immunosuppressants or in those with concurrent infection risk. Adverse effects include infusion reactions and rare thromboembolic events.

Tocilizumab (IL-6 receptor antagonist) is FDA-approved for several autoimmune and inflammatory indications, but its use in CIP is not established in the medical literature. Case reports and small series suggest potential benefits in other steroid-refractory irAEs and ongoing research is evaluating its role in pneumonitis.

Although data on these agents are limited in renal cell carcinoma, their use is supported by experience across tumor types and by mechanistic understanding of immune-mediated lung injury [103,107,108,109].

Patients receiving second-line immunosuppressive therapy require close monitoring for adverse effects including severe infections, hepatic dysfunction and hematologic abnormalities. Ideally within a multidisciplinary team involving pulmonologists, infectious disease and oncology.

### 6.5. Treatment Outcomes, Recurrence and Impact on Survival

Clinical outcomes in pneumonitis vary widely depending on severity at presentation: higher-grade pneumonitis (grade 3–4) is associated with increased morbidity, hospitalization, risk of permanent immunotherapy discontinuation, and inferior OS (HR for death 5.85 for higher-grade vs. lower-grade pneumonitis). Early recognition and prompt initiation of corticosteroids are associated with favorable outcomes, with approximately 80% of cases improving with guideline-directed management. Delayed diagnosis or severe presentation increases the risk of respiratory failure, the need for intensive care, and mortality. Recurrent pneumonitis occurs in up to 20–50% of patients who are rechallenged with immunotherapy, and higher BAL lymphocyte fractions or CD4/CD8 ratios > 1 have been associated with recurrence in some series [94].

### 6.6. Rechallenge Considerations in Renal Cell Carcinoma

Current recommendations for rechallenging ICI therapy after an episode of CIP in RCC emphasize individualized risk-benefit assessment and multidisciplinary decision-making. ASCO advises that rechallenge may be considered in select patients after complete resolution of pneumonitis, especially if the initial event was grade 1–2, rapidly responsive to corticosteroids, and if there is a compelling oncologic indication such as lack of alternative therapies or inadequate tumor response to initial therapy. Severe (grade 3–4) or steroid-refractory pneumonitis, persistent radiologic abnormalities, or significant residual pulmonary dysfunction are contraindications to rechallenge [32,95].

If rechallenge is pursued, rigorous monitoring protocols should be implemented. These include baseline high-resolution computed tomography, assessment of pulmonary function when feasible and close follow-up during the first several treatment cycles. Early reassessment is warranted at the onset of even mild respiratory symptoms. Some clinicians empirically use prophylactic low-dose corticosteroids, although evidence supporting this strategy is limited.

Ultimately, the decision to rechallenge must be individualized based on the patient’s clinical history, response to prior therapy, comorbidities and the availability of alternative treatments. The decision is best made within a multidisciplinary setting to balance oncologic benefit against potential respiratory risks.

**Table 2:** Diagnosis and management of ICI-related pneumonitis in RCC.

Step	Key Actions	Notes for RCC Patients	Reference
Initial suspicion	New respiratory symptoms or incidental CT abnormalities	Symptoms may overlap with TKI toxicity or metastases	[39,98]
High-resolution CT	Identify OP, NSIP, GGO, HP-like patterns	Compare with baseline, evaluate metastatic lesions	[38,51,60]
Bronchoscopy + BAL	Rule out infection, hemorrhage, tumor progression	Essential when fever, lymphopenia, or rapid decline are present	[91,92,97]
Differential diagnosis	Infection, pulmonary embolism, heart failure, TKI toxicity	TKI pulmonary effects can mimic CIP	[38,39,50,55,98]
Severity grading (ASCO, ESMO, SITC)	Grade 1 to Grade 4	Drives management decisions	[39,97]
Management—Grade 1	Hold ICI, monitor, repeat CT in 2–4 weeks	Resume ICI only if complete resolution	[39,97]
Management—Grade 2	Prednisone 1 mg/kg/day, taper 4–6 weeks	Hospitalize if worsening	[39,97,99]
Management—Grade ≥3	IV methylprednisolone, hospitalization, oxygen support	Consider ICU if hypoxic	[39,97,102,103,104,105,106,107,108,109]
Steroid-refractory cases	Infliximab, mycophenolate, IVIG, tocilizumab	Ensure infection is excluded before escalating	[96,103,104,105,106,107,108,109]
Outcomes and recurrence	Most improve, some relapse during taper	Severe cases may lead to permanent ICI discontinuation	[94,101,104]
Rechallenge	Only in selected Grade 1–2 cases fully resolved	Intensive monitoring, early CT re-evaluation	[95,96]

## 7. Future Perspectives

Despite significant advances in the management of RCC in the era of ICI–TKI combinations, several important knowledge gaps remain that limit our ability to accurately predict risk, optimize treatment, and tailor management strategies when immune-related toxicities occur. First, the true incidence and natural history of toxicities associated with ICI–TKI regimens in RCC are still incompletely characterized, largely because of heterogeneity in reporting and the absence of RCC-specific prospective registries. Most safety data come from mixed solid tumor cohorts or from experiences with ICI monotherapy, which do not adequately reflect the unique immunologic and vascular perturbations induced by combination treatments. Validated risk prediction models are also lacking. Clinical and laboratory factors used in other malignancies, such as NSCLC, have shown inconsistent associations in RCC, underscoring the need for disease-specific tools that incorporate the particular biology of renal tumors and the immunomodulatory effects of VEGF pathway inhibition. Optimal management strategies, especially concerning dose modifications, temporary treatment interruptions, and the safe timing of therapy rechallenge, also remain undefined and are often based more on expert consensus than on rigorous empirical evidence [96].

A major priority for future research is the identification and validation of reliable biomarkers capable of predicting susceptibility to treatment toxicity, distinguishing true immune-mediated events from injuries induced by TKIs, and guiding the safe reintroduction of therapy. Serologic markers such as C-reactive protein (CRP), Krebs von den Lungen-6 (KL-6), and circulating cytokines have been associated with pulmonary and systemic inflammatory responses in preliminary studies. Their dynamic monitoring might allow earlier detection of subclinical toxicity, better stratification of severity, and more accurate assessment of treatment response. However, their utility in RCC must be validated prospectively, as the baseline inflammatory milieu in these patients often differs from that of other tumor types because of tumor-related systemic effects and the influence of TKIs [14,104].

Imaging-based biomarkers also show considerable promise. Radiomic signatures derived from quantitative CT and MRI features may capture subtle parenchymal or microenvironmental alterations that precede clinical toxicity. Beyond enabling early diagnosis, radiomics could support the differentiation of immune-related pneumonitis from TKI-induced pneumonitis, infectious processes, or metastatic progression, all of which represent frequent diagnostic challenges in RCC. The integration of radiomics with clinical and serologic parameters may ultimately lead to multiparametric prediction models with improved accuracy [103,105,106].

Genomic and immunogenetic features are emerging as an additional frontier. Associations between specific HLA types and susceptibility to immune-related adverse events have been described in NSCLC and melanoma, although their relevance in RCC, particularly in patients treated with ICI–TKI combinations, remains to be elucidated. Tumor genomic alterations affecting antigenicity, interferon signaling, or immune-stromal interactions may further modulate the risk of toxicity and merit investigation in disease-specific cohorts.

Immune cell phenotyping in bronchoalveolar lavage (BAL) represents another promising approach, particularly in the differential diagnosis of pneumonitis. Insights gained from NSCLC have shown that characterizing T cell subsets, macrophage polarization, markers of exhaustion, and cytokine profiles can help distinguish immune-mediated pneumonitis from inflammation caused by TKIs or from infectious etiologies. BAL signatures could also act as functional readouts of ongoing immune activation, providing a level of precision that is difficult to achieve through peripheral blood analyses alone. Harmonized protocols for sample collection and flow cytometry would be necessary to ensure reproducibility across studies.

The substantial experience accumulated in NSCLC research offers a valuable framework for progress in the RCC field. In NSCLC, harmonized diagnostic criteria for immune-related adverse events have enabled consistent reporting across institutions and clinical trials. Standardized imaging follow-up schedules have facilitated the early detection of toxicities and improved their management. Prospective registries have provided high-quality data on incidence patterns, treatment responses, rechallenge outcomes, and long-term trajectories. Adapting similar infrastructures to RCC would help overcome current fragmentation and accelerate the generation of robust, disease-specific evidence.

Looking ahead, there is a major opportunity for translational and real-world research to refine management algorithms and guide rechallenge strategies. Prospective observational studies that incorporate longitudinal blood sampling, imaging, and tumor tissue analysis could systematically evaluate candidate biomarkers and support the development of predictive models. Complementary preclinical studies using RCC organoids, ex vivo immune assays, and murine models may clarify how VEGF inhibition reshapes immune responses and how these alterations interact with PD-1 or PD-L1 blockade at the tissue and cellular levels. Real-world data from multicenter registries and electronic health records will be essential to understanding toxicity patterns in diverse populations and to informing clinical decisions that extend beyond the constraints of clinical trials.

Another promising area is the development of clinical decision-support tools that integrate biomarkers, imaging results, and patient-specific risk profiles to guide individualized management. Such tools could help clinicians determine when bronchoscopy is warranted, when to escalate immunosuppression, and when rechallenge may be safe. They may also support the identification of patients who could benefit from alternative therapeutic agents or from reduced-intensity regimens designed to minimize toxicity without compromising antitumor activity.

In summary, progress in the management of toxicities associated with ICI–TKI combinations in RCC will depend on closing disease-specific knowledge gaps, developing and validating multimodal biomarkers, and incorporating insights drawn from more mature fields such as NSCLC. Through coordinated translational, clinical, and real-world research efforts, it will be possible to refine risk prediction models, personalize treatment, and ultimately enhance both the safety and efficacy of combination therapy in RCC.

## 8. Conclusions

CIP is an underrecognized yet clinically meaningful toxicity in patients with RCC with ICI alone or in combination with TKIs. Although the incidence reported in clinical trials is relatively low, emerging real-world evidence suggests that CIP may be more common and more heterogeneous than previously appreciated. Its clinical relevance is amplified by the potential for rapid progression, substantial morbidity, and the possibility of permanent treatment discontinuation, all of which can significantly influence long-term oncologic outcomes.

In RCC, the diagnostic process is particularly challenging. Disease-related pulmonary abnormalities, treatment-induced radiologic changes, infectious complications, and metastatic involvement frequently overlap, making CIP difficult to distinguish from alternative etiologies. This intrinsic complexity contributes to underreporting, delayed diagnosis, and inconsistency in therapeutic decision-making. The absence of RCC-specific diagnostic pathways and validated biomarkers further complicates clinical evaluation and often results in reliance on expert judgment rather than standardized criteria. As ICI–TKI combination regimens increasingly become the therapeutic backbone for advanced RCC, the diagnostic ambiguity surrounding pulmonary toxicities represents a growing unmet need.

Improving patient outcomes will require earlier recognition of subtle clinical and radiologic signs, closer multidisciplinary coordination between oncologists, pulmonologists, radiologists, and immunologists, and more systematic approaches to evaluation and follow-up. The integration of structured imaging review, predefined diagnostic algorithms, and greater familiarity with CIP presentations will help reduce variability in management. Equally important is the expansion of dedicated research efforts, including prospective registries, translational studies exploring radiomic, serologic, and immunologic markers, and real-world analyses aimed at clarifying incidence patterns and predictors of severity. These initiatives have the potential to refine risk stratification, support timely intervention, and inform safe strategies for treatment rechallenge.

Recognizing CIP as a clinically significant challenge in RCC is a crucial step toward improving patient care. A combination of heightened clinical vigilance, standardized diagnostic processes, and robust research programs will be essential to reduce uncertainty, optimize therapeutic choices, and ultimately enhance both the safety and effectiveness of modern immunotherapy-based treatments for RCC.

## Figures and Tables

**Table 1:** Incidence of immune checkpoint inhibitor-related pneumonitis (CIP) in pivotal trials of advanced RCC.

Trial	Regimen		Comparator	All-Grade CIP	Grade ≥ 3 CIP	Regimen Class	Key Limitations	Reference
CheckMate 214	Nivo + Ipi	547	Sunitinib	6–7%	1–2%	Dual ICI	Possible under-reporting; selected population	[7]
CheckMate 9ER	Nivo + Cabo	323	Sunitinib	3–4%	<1%	ICI–TKI	Difficult distinction from TKI toxicity	[6]
KEYNOTE-426	Pembro + Axi	432	Sunitinib	2–4%	<1%	ICI–TKI	Trial population healthier than real world	[5]
JAVELIN Renal 101	Ave + Axi	442	Sunitinib	1–3%	<1%	ICI–TKI	Potential under-ascertainment	[18]
CLEAR	Pembro + Lenva	355	Sunitinib	1–3%	<1%	ICI–TKI	Radiologic review not centralized	[4]
CheckMate 025	Nivolumab	406	Everolimus	3%	<1%	PD-1 monotherapy	Limited external validity	[19]

## Data Availability

No new data were created or analyzed in this study. Data sharing is not applicable to this article.
